# Magnetic Resonance pH Imaging in Stroke – Combining the Old With the New

**DOI:** 10.3389/fphys.2021.793741

**Published:** 2022-02-03

**Authors:** James R. Larkin, Lee Sze Foo, Brad A. Sutherland, Alexandre Khrapitchev, Yee Kai Tee

**Affiliations:** ^1^Department of Oncology, Medical Research Council Oxford Institute for Radiation Oncology, University of Oxford, Oxford, United Kingdom; ^2^Lee Kong Chian Faculty of Engineering and Science, Universiti Tunku Abdul Rahman, Kajang, Malaysia; ^3^Tasmanian School of Medicine, College of Health and Medicine, University of Tasmania, Hobart, TAS, Australia

**Keywords:** magnetic resonance spectroscopy, chemical exchange saturation transfer, magnetic resonance imaging, pH, stroke, ^31^P

## Abstract

The study of stroke has historically made use of traditional spectroscopy techniques to provide the ground truth for parameters like pH. However, techniques like ^31^P spectroscopy have limitations, in particular poor temporal and spatial resolution, coupled with a need for a high field strength and specialized coils. More modern magnetic resonance spectroscopy (MRS)-based imaging techniques like chemical exchange saturation transfer (CEST) have been developed to counter some of these limitations but lack the definitive gold standard for pH that ^31^P spectroscopy provides. In this perspective, both the traditional (^31^P spectroscopy) and emerging (CEST) techniques in the measurement of pH for ischemic imaging will be discussed. Although each has its own advantages and limitations, it is likely that CEST may be preferable simply due to the hardware, acquisition time and image resolution advantages. However, more experiments on CEST are needed to determine the specificity of endogenous CEST to absolute pH, and ^31^P MRS can be used to calibrate CEST for pH measurement in the preclinical model to enhance our understanding of the relationship between CEST and pH. Combining the two imaging techniques, one old and one new, we may be able to obtain new insights into stroke physiology that would not be possible otherwise with either alone.

## Introduction

Acute ischemic stroke causes a reduction in blood supply to the brain, resulting in the deprivation of energy. Unless rapidly reversed, prolonged ischemia can lead to cerebral infarction. Thrombolysis or recanalization therapy is the main treatment to salvage the tissue that is still viable through timely reperfusion; the salvageable tissue is termed ischemic penumbra. The conventional methods to estimate the penumbra are through the spatial mismatch between the ischemic core and the hypoperfused area, e.g., *via* computed tomography (CT) and CT perfusion imaging (Lin and Liebeskind, 2016), as well as diffusion and perfusion magnetic resonance imaging (MRI; Demeestere et al., 2020). However, this estimation of the penumbra is often inaccurate, falsely including regions of benign oligemia that will recover irrespective of treatment (Leigh et al., 2018).

When ischemia happens, the normal intracellular pH level of around pH 7.2, regulated by active and passive mechanisms (Orlowski et al., 2011; Jaworska et al., 2021), begins to drop. The depletion of oxygen and metabolic substrates triggers anaerobic glycolysis accompanied by a build-up of lactate, resulting in tissue acidosis (Durukan and Tatlisumak, 2007). Tissue partial pressure of carbon dioxide increases, contributing to the acidification of the tissue (Orlowski et al., 2011). In the very center of the infarct, the ischemic core, tissue pH may decrease to as low as 6.0, while in the penumbra, pH level ranges between 6.5 and 6.9, as demonstrated in animal stroke models (Tóth et al., 2020a). Tissue acidosis may subsequently induce cellular damage through the production of free radicals, impaired protein synthesis, mitochondrial failure, DNA damage, and accumulation of calcium ions (Durukan and Tatlisumak, 2007; Radak et al., 2017). Thus, having real-time spatial pH information could provide clinically useful information in the diagnosis and treatment of ischemic stroke, such as the estimation of the “real” penumbra (Harston et al., 2015) or potential targeted delivery of neuroprotective agents (Tóth et al., 2020a), leading some to suggest penumbra may be better estimated through pH imaging instead of spatial mismatch of CT and CT perfusion or diffusion and perfusion MRI (Heo et al., 2017; Kim et al., 2021).

Magnetic resonance spectroscopy (MRS) is a non-invasive technique that allows for the measurement of different metabolic or neurochemical profiles of the brain *via* the spectra of atomic nuclei such as ^1^H, ^13^C, ^19^F, ^23^Na, or ^31^P (Cichocka et al., 2015). Historically ^31^P MRS, whose signal originates from inorganic phosphate (Pi), adenosine triphosphate, adenosine diphosphate, phosphocreatine (PCr), and sugar phosphates (Gorenstein and Luxon, 2017), has long been regarded as the gold standard for the measurement of intracellular pH. However, many aspects of ^31^P spectroscopy still limit the use of the technique in the clinical setting, mostly arising from relatively poor spatial and temporal resolution, as well as the need for high field strengths and specialized receiver coils. More recently, an MRI technique known as chemical exchange saturation transfer (CEST), that allows for the detection of low-concentration metabolites, has also shown promise in providing quantitative pH information. However, CEST does lack the association with the definitive gold standard provided by MRS as the effect is not only dependent upon pH, but also dependent upon concentration and water relaxation.

The first journal article which showed ^31^P spectroscopy could be used to measure intracellular pH was published in Moon and Richards (1973) whereas the first journal article using the name CEST was published in Ward et al. (2000). However, it was only in Zhou et al. (2003) managed to show CEST could be used to measure intracellular pH in a preclinical stroke model. Nevertheless, proposing chemical exchange as one of the transfer of magnetization mechanisms was brought up well before the first CEST paper. Here, we have regarded ^31^P spectroscopy as the “old” technique and CEST as the “new” technique, based on when the terms were first used.

This perspective briefly discusses the advantages and limitations of both the traditional (^31^P MRS) and emerging nuclear magnetic resonance technique (CEST) in the measurement of pH for ischemic stroke investigation, and subsequently makes recommendations for potential clinical measurement of pH in stroke.

## Phosphorus Magnetic Resonance Spectroscopy, ^31^P

Spectroscopy using ^31^P is very straight-forward data acquisition approach with a single excitation pulse and a direct readout of ^31^P spectra. The measurement of pH using ^31^P MRS is possible as there are peaks within the ^31^P spectrum of tissue that have a chemical shift dependent upon pH. These changes in chemical shift arise because one of the pK_*a*_ values for Pi is found in the physiologically relevant range, specifically the pK_*a*_ for the H_2_PO_4_^–^ ⇌ HPO_4_^2–^ + H^+^ equilibrium is 7.20. This means that the chemical shift for the Pi peak is sensitive to pH (Moon and Richards, 1973), whereas the peaks corresponding to other phosphate-containing species such as PCr are relatively insensitive to pH changes. Thus, through the measurement of the chemical shift difference between Pi and PCr, intracellular pH can be estimated through the modified Henderson–Hasselbalch equation (Seo et al., 1983; Kim et al., 2021):


(1) $$\text{pH}={\text{pK}}_{\text{a}}+\text{log}\,\left[\frac{\delta -\delta_{\text{acid}}}{\delta_{\text{base}}-\delta }\right]=6.77+\log\,\left[\frac{\delta -3.29}{5.68-\delta }\right]$$

where δ is the difference between the observed chemical shifts, δ_*acid*_ and δ_*base*_ are the chemical shifts of the acid and its conjugate, respectively, and pK_a_ is the negative log of the acid base equilibrium constant. There are two sets of coefficients for Eq. (1) that are both commonly used in the brain pH literature (Cichocka et al., 2015). Although each set of coefficients results in minor differences of pH of up to ∼0.03 pH units, there is no consensus on which is better. In this perspective, we used pK_a_ = 6.77, δ_*acid*_ = 3.29 and δ_*base*_ = 5.68.

Due to its specificity, reliability, and extensive validation with microelectrode measurements, ^31^P MRS has long been regarded as the gold standard for *in vivo* and non-invasive pH measurement (Kim et al., 2021) although pH can also be evaluated with ^1^H MRS *via* tissue lactate concentration which has a linear correlation with pH (Jokivarsi et al., 2007). Unfortunately, ^31^P has several key limitations that prevent its widespread clinical use.

Firstly, ^31^P resonates at a different frequency to ^1^H which means that ^31^P MRS requires different MR coils and software, including acquisition pulse sequences, reconstruction algorithms, and post-reconstruction analyses, that are not commonly found in conventional clinical MRI machines (Novak et al., 2014). In fact, the United States Food and Drug Administration (FDA) has cleared only a handful of MRI coils compatible with ^31^P acquisitions (510(k) Premarket Notification Database). Furthermore, depending on coil design, the use of a dedicated transmit or receive coil for ^31^P acquisition may preclude the simultaneous acquisition of ^1^H data. This lack of general availability of both hardware and software furthermore means that the knowledge required to undertake ^31^P acquisitions is often lacking in radiographers. This means that even if hardware and software problems are solved, only centers with specialist staff would be able to acquire ^31^P data. Beyond these hardware, software, and personnel limitations, ^31^P acquisitions also have physical limitations such as their very poor signal strength, leading to a poor signal-to-noise ratio (SNR). Eq. (2) shows SNR is dependent upon the number of molecules in the observed sample (N; concentration), the abundance of NMR-active spins (A; 100% for ^31^P), the temperature (T) of the sample, the magnetic field strength (B_0_), the gyromagnetic ratio of the observed nucleus (γ), the effective transverse relaxation time (T_2_*), and the number of signal averages (NSA; Claridge, 2016). Of these, the most critical for driving the low SNR in *in vivo* ^31^P acquisitions is the low concentration of the relevant ^31^P-containing species: the concentration of most species is on the order of a few mM compared to the multi-molar concentrations of ^1^H-containing species; around 70–80% of our body consists of water and fat that have abundant ^1^H protons (over 100 M proton concentration in total). Also important is the lower gyromagnetic ratio of ^31^P (17.2 MHz/T) as compared to ^1^H (42.6 MHz/T), which manifests as 9.7-fold reduction in SNR, all other factors being equal. We can partially compensate for the low SNR by using high-field MRI systems (e.g., 7.0 or 9.4 T, compared to a conventional 1.5 or 3 T system), but this does not yield substantial improvements.


(2) $$\text{SNR}\propto N\cdot A\cdot T^{-1}\cdot B_{0}^{3/2}\cdot \gamma^{5/2}\cdot T_{2}^{*}\cdot \sqrt{N\,S\,A}$$

This poor inherent SNR with ^31^P manifests as low spatial resolution (about 20 ml) and low temporal resolution. At a typical clinical MRI field strength of 1.5–3 T, as well as the SNR limitations discussed, ^31^P acquisitions are further complicated by spectral overlaps between different species (Wu et al., 2016).

The approach of acquiring many averages to boost SNR can massively prolong acquisition times, especially if hundreds or thousands of averages are acquired. In some diseases, like cancer, increasing the required number of averages is time-consuming, but not insurmountable. Likewise, some dynamic situations lend themselves to repeated averaging if the tissue can be “reset” between each acquisition. One example would be studying pH changes in muscle during exercise, where sufficient rest periods allow repeated acquisitions on the same volume of tissue (Forbes et al., 2009). In a condition like stroke, with rapid and irreversible changes to tissue pH, it is not possible to increase the number of averages in a conventional fashion. Owing to the dynamic nature of the infarct, increasing the number of free induction decays (FIDs) included in an average simply results in a signal representing the mean physiological state during acquisition, but does not necessarily offer biological insight. Often, this mean physiological state spectrum cannot even be used to determine a mean pH, as the movement of the Pi peak during the acquisition leads to it being lost in the noise. One other method for boosting SNR is to increase the voxel size for acquisitions, with signal increasing in proportion to volume. Although this approach is feasible, and indeed common, when analyzing tissue like muscle, it has less utility with stroke, where mean infarct volume is commonly often far under 200 mL (Hug et al., 2009; Zaidi et al., 2012), with considerably smaller cores, limiting the extent that signal can be increased.

One approach to overcome these limitations in experimental conditions is to use a “rolling-average” approach to data acquisition. ^31^P data is acquired in a series of FIDs, each around 3 s long. Normally a long acquisition of ^31^P data would sum many of these FIDs into a single dataset. Although a single FID has a very poor SNR, when they are summed it is possible to provide sufficient SNR for analysis, at the expense of temporal resolution. The rolling-average approach described here stores each low-SNR FID separately, giving the option to create a rolling-average post-acquisition. Here we combine 300 FIDs, representing 15 min of acquisition into a single rolling average window, before moving one FID along the data set and repeating the averaging process. Thus, the first average window includes FIDs 1–300 centered around FID 150, the second average window includes FIDs 2–301, the third includes FIDs 3–302 etc. In this way the temporal resolution is kept high (3 s) while still allowing sufficient averaging to achieve the required increase in SNR. The number of FIDs in the rolling average window is a tunable variable that must be adjusted to achieve a compromise between having sufficient sequential FIDs for adequate SNR, but few enough that the change of pH within the average duration does not lead to excessive blurring of the Pi peak as it moves.

**FIGURE 1:**
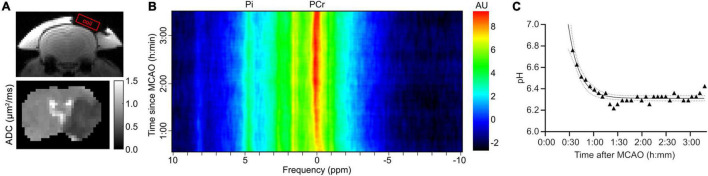
Example of an estimation of pH over time using a rolling average acquisition in an experiment in a male SD rat that underwent ^31^P spectroscopy after a MCAO. **(A)** The position of spectroscopy coil and apparent diffusion coefficient (ADC) map (μm^2^/ms) of the rat brain, showing the extent of the infarct. **(B)** Image map showing rolling average spectral data against time after MCAO, with the positions of the PCr and Pi peaks indicated. Note the decreasing separation of the two peaks as the time increases. Each rolling average period is 300 FIDs, or 15 min. **(C)** Scatter plot of pH calculated according to Eq. (1) against time after MCAO. Solid line is a fitted one-phase exponential decay with 99% confidence intervals indicated by the dashed lines.

Figure 1 shows an example of how this rolling-average acquisition looks in an animal stroke model, with a rolling-average window of 15 min. The change in pH across time post middle cerebral artery occlusion (MCAO) was estimated through the Pi and PCr chemical shifts using Eq. (1); a rapid decrease in pH after stroke was observed followed by a plateau. The initial decrease, between 34 and 61 min after MCAO was approximately 0.39 pH units, corresponding to a decrease of approximately 0.014 pH units/min. The pH settles at a plateau at pH 6.31 (99% confidence intervals: 6.29–6.33) after 1–2 h. A single multi-FID ^31^P acquisition would not reveal this detail.

Recently, ^31^P MRS/MRS imaging (MRSI) has also been increasingly studied at ultra-high field strengths (≥7 T) in different applications, such as metabolic neuroimaging of Parkinson’s patients (Zhu et al., 2021), amnestic mild cognitive impairment (Das et al., 2020), brain tumor imaging (Mirkes et al., 2016), and cardiac MRS (Ellis et al., 2019). It would be interesting to see future investigations on the use of ^31^P MRS in stroke at these high field strengths as well.

## Chemical Exchange Saturation Transfer Magnetic Resonance Imaging

**FIGURE 2:**
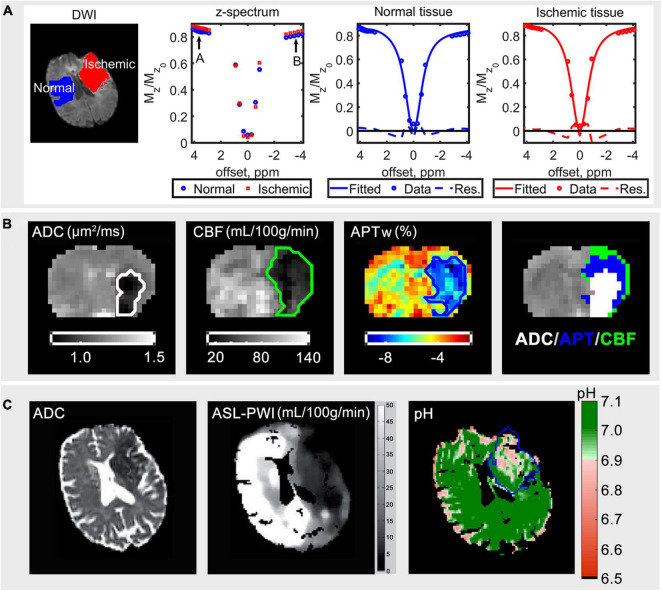
**(A)** Diffusion weighted imaging (DWI) data for *b* = 1,000 s/mm^2^ of an ischemic stroke patient, the average acquired z-spectrum of normal and ischemic tissue, fitted spectrum in the normal and ischemic tissue from left to right, modified from Tee et al. (2014). **(B)** The ADC (μm^2^/ms), cerebral blood flow (CBF) (mL/100 g/min), APT-weighted (APWw) (%) images, and ADC/APT/CBF mismatch of an animal that underwent middle cerebral artery occlusion; the order of display priority of the mismatch map is white (ADC) > blue (APT) > green (CBF). **(C)** The ADC, arterial spin labeling perfusion weighted imaging (ASL-PWI) (mL/100 g/min) (Harston et al., 2015) and quantitative pH maps of a hyperacute stroke patient; the blue line outlines the infarct core defined using diffusion-weighted imaging (Tee et al., 2014). The figures are reproduced with permission from the publishers.

Unlike ^31^P MRS, CEST MRI measures the magnetization signal of water protons, ^1^H, directly through the chemical exchange of the saturated solute protons to water protons (Ward and Balaban, 2000; Ward et al., 2000). Although the solute protons have a low concentration (mM), the signal of these solute protons can be amplified by >100 times *via* the build-up of saturation of water in CEST MRI (Wu et al., 2016). This can lead to a CEST effect (a dip) at the resonance frequency of the solute protons when the measured saturated water proton signal is plotted across different frequency offsets, commonly known as the z-spectrum. An example of a normal and ischemic z-spectrum of an ischemic stroke patient are shown in Figure 2A.

Magnetization transfer asymmetry analysis is the most commonly used method to quantify the CEST effect, defined as:


(3) $${\text{MTR}}_{\text{asym}}\,\left(△\,\omega \right)\,\frac{M_{z}\,\left(-△\,\omega \right)-M_{z}\,\left(+△\,\omega \right)}{M_{z\,0}}$$

where *M*_*z*_ ( △ω) refers to the measured signal at frequency offset, △ω and *M*_*z0*_ is the unsaturated signal, basically taking the value pointed by label B minus the value pointed by label A in Figure 2A. There are also other quantification methods such as fitting the acquired z-spectrum to the multi-Lorentzian lineshape function or the modified Bloch McConnell equations to quantify the CEST effect as presented in Figure 2A. For details about the most commonly used quantification methods of CEST in stroke and their definitions, readers are referred to (Foo et al., 2021b).

A variant of CEST investigating the transfer of saturation from amide protons to water resonating at +3.5 ppm, termed amide proton transfer (APT) was found to be sensitive to intracellular pH as the exchange rate of amide protons is base-catalyzed at physiological pH range (Zhou J. et al., 2019). It was shown that the exchange rate of amide protons had a high correlation with pH, confirmed by ^31^P MRS at normocapnia and post-mortem. By forming a relationship between the amide proton transfer ratio, APTR, and pH, high resolution quantitative measurement of intracellular pH map was made possible through use of Eq (4), Zhou et al. (2003).


(4) $$\text{APTR}=5.73\times 10^{\text{pH}-6.4}$$

Indeed, animal studies have demonstrated APT coupled with diffusion weighted imaging and perfusion weighted imaging to have the potential of delineating the ischemic injury into three zones, possibly: the infarct core, the acidotic penumbra, and the benign oligemia (Foo et al., 2021b), as shown in the representative results of a rat brain that underwent MCAO (Figure 2B). The high-resolution quantitative pH map had also been demonstrated in a clinical study (Tee et al., 2014), where the pH value was shown to be lower in the ischemic area as shown in Figure 2C.

Compared to ^31^P MRS, CEST MRI is able to provide a higher spatial image resolution. In ^31^P MRS, pH information is usually acquired from a region of interest *via* multiple averages (Figure 1B) or more recently with MRSI to acquire pixel-wise pH information using ultrahigh field strength scanners (7 T and above). However, because the *in vivo* concentration of Pi is very low and thus the SNR, the ^31^P MRSI acquisition time has to be long (tens of minutes) to ensure a reliable detection of the Pi resonance for cubic centimeter range spatial resolution (Korzowski et al., 2020). Whereas it is possible to acquire pixel-wise pH information in the cubic millimeter range spatial resolution under 5 min *via* CEST MRI in the clinical field strength scanners such as 3 T (Tee et al., 2014; Figure 2C).

The measurement of signal from water protons instead of ^31^P also puts CEST in an advantage in terms of sensitivity and SNR due to the abundance of water and fat in the body. Additionally, there is also a hardware advantage as most MRI scanners in hospitals are predominantly ^1^H MRI scanners, meaning it is possible to acquire the pH information with no additional hardware needed when using CEST.

Furthermore, CEST imaging can be used to measure extracellular pH when clinically approved X-ray contrast agents such as iopamidol and iobitridol are used. Highly quantitative extracellular pH maps have been successfully produced in preclinical tumor imaging (Anemone et al., 2017). Although the use of this in ischemic stroke may be limited due to the nature of the disease, the potential of measuring both the intra- and extra-cellular pH using a single imaging sequence with clinically approved contrast agents can lead to a shorter development time and thus faster translation. The better image resolution, higher SNR and lower technological barrier to entry have led to a high interest in using CEST for clinical stroke imaging (Foo et al., 2021a), competing with ^31^P MRS.

Despite the demonstration of the capability of CEST to measure absolute pH in either intra- or extra-cellular space, this imaging technique has its limitations. In particular, the relationship between the APT signal and pH only holds true in the hyperacute stage as assumptions such as negligible amide and water content changes are made to form Eq. (4). In the subacute and chronic stroke stages, this established relationship is no longer valid because changes in amide proton concentration as well as water relaxation times are common in these stages which can confound the APT signal. Although the quantified APT effect in these stages is likely to be the composite changes of all the mentioned factors, it is still found to have significant inverse correlation with the National Institutes of Health Stroke Scale (NIHSS) and 90-day modified Rankin Scale (mRS) scores, highlighting its potential in assessing stroke severity and predicting clinical outcome of acute stroke patients imaged after 24–48 h from symptoms onset (Lin et al., 2018). It has also been proposed as a biomarker of ischemic stroke recovery in patients receiving supportive treatment, where the increase in quantified APT effects post-treatment was associated with clinical symptom improvements while the opposite was observed in patients exhibiting aggravated symptoms (Yu et al., 2019).

Another issue of CEST MRI is that both the concentration and exchange rate share a similar effect on the CEST signal, meaning either one of the parameters can cause a change to the APTR in Eq. (4) but only the exchange rate is pH dependent. Apart from the amide protons which resonate at 3.5 ppm downfield from the water resonance, the measured APTR is also affected by the neighboring exchangeable protons such as amine protons which resonate from 2 to 3 ppm. Depending on the experimental parameters used in CEST imaging, the measured APTR has been found to be minimally to significantly affected by the neighboring protons (Zhang et al., 2018).

When CEST is used in higher field strength clinical scanners (7 T), the magnetic field inhomogeneity is found to be higher and it is harder to maintain homogenous radiofrequency pulses across the whole brain. Nevertheless, this is less of an issue at the moment because the majority of clinical scanners are 3 T and below, and so far all the published studies assessing APT in ischemic stroke patients up to 31st December 2020 used 3 T scanners and most of them used single slice acquisition (Foo et al., 2021a).

Chemical exchange saturation transfer imaging can be performed using a continuous pulse or multiple short pulses denoted as continuous and pulsed saturation, respectively. The former is commonly used in preclinical experiments and is more efficient, resulting in smaller spillover effect to the neighbor offsets. However, pulsed saturation is preferred in humans although it has a higher spillover effect due to the radio frequency amplifier and specific absorption rate limitation. Denoising the acquired z-spectrum by either fitting a model (Lorentzian or modified Bloch-McConnell equations) or interpolation based smoothing algorithms has been found to improve the reproducibility and robustness of the CEST data to noise (Foo et al., 2020). However, using an unsuitable model or algorithm to describe the different broad and slowly varying pools to fit or denoise the z-spectrum can lead to errors. Besides that, a few volumetric CEST imaging techniques are developed to cover more slices and frequency offsets by sacrificing SNR of the acquired data (Cai et al., 2019), this has posed significant challenges to fitting the acquired spectra for more robust quantification.

A few solutions have been proposed to mitigate some of the mentioned limitations in CEST MRI for pH imaging. For separating the exchange rate effect from the concentration in APTR, multi radiofrequency saturation schemes have been found useful (Wu et al., 2015; Sun, 2021). Likewise, the change of water relaxation time during stroke can be compensated by obtaining quantitative water relaxation maps (Zhou I.Y. et al., 2019; Foo et al., 2021b). The initial success of CEST MRI in the measurement of pH for ischemic stroke has shown some promise although some obstacles remain to prevent it from becoming a routine clinical stroke imaging modality. This is an interesting topic that is still under active research because pH has long been regarded as an important biomarker for ischemic stroke management. Recently, there are some papers reporting the use of CEST for lactate detection (DeBrosse et al., 2016; Lenich et al., 2018; Saito et al., 2019), it will be interesting to see how this can be added to other known proton exchanges at different frequency offsets for stroke diagnosis in the future.

## Discussion

Both ^31^P and CEST MRI have distinct advantages and disadvantages, each offering insights into pH in stroke in specific ways. Although it would be ideal to have the gold-standard ^31^P data available under all circumstances, physics and practical limitations prevent this. The American Heart Association (AHA) recommends that stroke imaging should be performed within 25 min of emergency department (ED) arrival and the results should be ready within 45 min of ED arrival (Ringleb et al., 2008). It is infeasible to acquire ^31^P and/or CEST data on top of all the recommended scans for acute stroke patients. However, acquiring information regarding pH levels following stroke could have important implications for diagnosis and management.

A pH change in brain tissue following stroke correlates well with the level of ischemia experienced by that tissue, considering differences in pH between the ischemic core and penumbra have been found (Sun et al., 2007; Harston et al., 2015). In addition, assessment of not only the extent of pH change but the rate of pH change could provide an indication of metabolic health of the tissue with changes in lactate and partial pressure of carbon dioxide contributing to acidosis. Tissue that leads to an equilibrium or recovery in pH could predict overall tissue recovery. Also, the acidosis in ischemic tissue has been recently utilized for therapeutic strategies, with acidosis-responsive nanoparticles being able to release neuroprotectants (Tóth et al., 2020b). Understanding the temporal dynamics of pH change post-stroke will enable improved therapeutic targeting in the right location and time window for ischemic stroke.

It is for these two reasons (limited imaging time available and the importance of pH in stroke) that we propose combining ^31^P and CEST acquisitions in preclinical models, and then using the lessons learned regarding pH dynamics to apply CEST primarily in human patients. The rolling-average technique described herein can offer definitive pH data in experimental stroke systems. By using the rolling average method, sensitivity can be increased because useful averaging can be obtained over a wider temporal window. This increased insight into real-time pH in the preclinical model obtained by the gold-standard ^31^P can then be combined with the preclinical CEST data. Acquiring CEST data in the same experimental system along with the rolling-average ^31^P data can give us a high confidence over physical conditions during the experiment. This could be used to separate the effect of amide proton exchange rate from its concentration. Consequently, it may become possible to monitor pH evolution after stroke (while the concentration is stable) and also molecular level changes such as cell membrane breakdown (while the pH is constant). Combining both the pH imaging techniques in the preclinical setting may provide new insights into stroke pathophysiology that may revolutionize how acute stroke patients are diagnosed, managed and treated in the future.

## Conclusion

It is possible that ^31^P and/or CEST MRI could be translated into routine clinical stroke management in the future. However, it is likely that CEST MRI would have the upper hand simply due to the hardware, acquisition time and image resolution advantages. Ultimately, more experiments on CEST are needed to determine the specificity of endogenous CEST to absolute pH, and ^31^P MRS can be used to calibrate CEST for pH measurement in preclinical models to enhance our understanding of CEST. By combining the two magnetic resonance pH imaging methods, one old and one new, we may be able to obtain new insights into stroke pathophysiology that would not be possible otherwise with either alone.

## Data Availability Statement

The raw data supporting the conclusions of this article will be made available by the authors, without undue reservation.

## Ethics Statement

The studies involving human participants were reviewed and approved by the United Kingdom National Research Ethics Service Committee South Central (ref: 12/SC/0292). The patients/participants provided their written informed consent to participate in this study. Animal experiments were authorised by the United Kingdom Home Office and conducted in accordance with the University of Oxford Policy on the Use of Animals in Scientific Research.

## Author Contributions

JL, BS, and AK conducted the experiments. JL, LF, and AK analyzed the data. JL, LF, BS, and YT wrote the manuscript. All authors read and approved the manuscript.

## Conflict of Interest

The authors declare that the research was conducted in the absence of any commercial or financial relationships that could be construed as a potential conflict of interest.

## Publisher’s Note

All claims expressed in this article are solely those of the authors and do not necessarily represent those of their affiliated organizations, or those of the publisher, the editors and the reviewers. Any product that may be evaluated in this article, or claim that may be made by its manufacturer, is not guaranteed or endorsed by the publisher.
